# Fingolimod effects on the brain are mediated through biochemical modulation of bioenergetics, autophagy, and neuroinflammatory networks

**DOI:** 10.1002/pmic.202100247

**Published:** 2022-08-08

**Authors:** Mehdi Mirzaei, Morteza Abyadeh, Anita J. Turner, Roshana Vander Wall, Joel M. Chick, Joao A. Paulo, Veer K. Gupta, Devaraj Basavarajappa, Nitin Chitranshi, Seyed Shahab Oddin Mirshahvaladi, Yuyi You, Matthew J. Fitzhenry, Ardeshir Amirkhani, Paul A. Haynes, Alexander Klistorner, Vivek Gupta, Stuart L. Graham

**Affiliations:** ^1^ Department of Clinical Medicine Faculty of Medicine Health and Human Sciences Macquarie Medical School Macquarie University Macquarie Park, North Ryde Sydney NSW Australia; ^2^ ProGene Technologies Pty Ltd Sydney NSW Australia; ^3^ Department of Cell Biology Harvard Medical School Boston Massachusetts USA; ^4^ School of Medicine Deakin University Geelong VIC Australia; ^5^ Australian Proteome Analysis Facility Macquarie University Sydney NSW Australia; ^6^ School of Natural Sciences Macquarie University Macquarie Park NSW Australia; ^7^ Biomolecular Discovery Research Centre Macquarie University Sydney NSW Australia

**Keywords:** Fingolimod, neurodegeneration, neuroinflammation, oxidative phosphorylation, quantitative proteomics

## Abstract

Fingolimod (FTY720) is an oral drug approved by the Food and Drug Administration (FDA) for management of multiple sclerosis (MS) symptoms, which has also shown beneficial effects against Alzheimer's (AD) and Parkinson's (PD) diseases pathologies. Although an extensive effort has been made to identify mechanisms underpinning its therapeutic effects, much remains unknown. Here, we investigated Fingolimod induced proteome changes in the cerebellum (CB) and frontal cortex (FC) regions of the brain which are known to be severely affected in MS, using a tandem mass tag (TMT) isobaric labeling‐based quantitative mass‐spectrometric approach to investigate the mechanism of action of Fingolimod. This study identified 6749 and 6319 proteins in CB and FC, respectively, and returned 2609 and 3086 differentially expressed proteins in mouse CB and FC, respectively, between Fingolimod treated and control groups. Subsequent bioinformatics analyses indicated a metabolic reprogramming in both brain regions of the Fingolimod treated group, where oxidative phosphorylation was upregulated while glycolysis and pentose phosphate pathway were downregulated. In addition, modulation of neuroinflammation in the Fingolimod treated group was indicated by upregulation of retrograde endocannabinoid signaling and autophagy pathways, and downregulation of neuroinflammation related pathways including neutrophil degranulation and the IL‐12 mediated signaling pathway. Our findings suggest that Fingolimod may exert its protective effects on the brain by inducing metabolic reprogramming and neuroinflammation pathway modulation.

**Abbreviations**
AD: Alzheimer's disease
CB: cerebellum
CNS: central nervous system
FC: frontal cortex
MS: multiple sclerosis
NfL: Neurofilament light chain
PD: Parkinson's disease
S1PR: sphingosine 1‐phosphate receptor
TMT: tandem mass tag

## 1 INTRODUCTION

Fingolimod (FTY720) is the first orally administered disease modifying agent approved by the Food and Drug Administration (FDA) for management of multiple sclerosis (MS) [1, 2]. Fingolimod is approved as a second‐line treatment drug for relapsing remitting MS in the European Union and Canada and as a first‐line treatment drug in the United States and Australia [3, 4]. Fingolimod is an analog of the endogenous sphingosine‐1 phosphate which binds to sphingosine 1‐phosphate receptors (S1PRs) including S1P1, 3, 4, and 5, and affects their activity [5]. S1PRs are a class of G protein‐coupled receptors that are expressed by a wide range of cells in different organs such as brain, retina, heart, liver, and stomach [6]. Within the brain, these receptors are expressed on different cells including neurons, oligodendrocytes, astrocytes, and microglia [5]. In MS, these receptors have been shown to promote neuroinflammation and subsequent neurodegeneration through increasing activation and proliferation of astrocytes, microglia, and central nervous system (CNS)‐infiltrating proinflammatory monocytes [5, 6, 7, 8]. Fingolimod can cross the blood–brain barrier (BBB) and affect S1PR receptors in the CNS [9]. Although little is known about the mechanisms underlying therapeutic effects of Fingolimod in MS patients, currently the main hypothesis is that it exerts anti‐inflammatory properties by binding to S1PRs and inhibiting subsequent neuroinflammation [10].

Neuroinflammation is the underlying pathology in several neurodegenerative diseases such as Alzheimer's (AD) and Parkinson's (PD) diseases [11, 12]. To date, a limited number of studies have been performed to investigate the potential beneficial effects of fingolimod as an anti‐inflammatory agent in the context of these diseases. The drug has yielded promising results and indicated both anti‐inflammatory and neuroprotective effects in various pre‐clinical studies [13, 14]. Although some insights into the therapeutic mechanisms of Fingolimod actions in the brain have been identified, such as an increase in level of brain‐derived neurotrophic factor (BDNF) in PD animal models or activation of the Caspase‐3 pathway and reduction of Aβ concentration through inhibiting beta‐secretase (BACE) and ceramide in AD animal models [13, 15], the complete mechanism of action of the drug remains to be determined. High throughput technologies such as genomics, transcriptomics, proteomics, metabolomics, and lipidomics have enabled us to study the complete set of disease associated changes at different molecular levels and opened a new window for disease investigation to fuel the discovery of new therapeutics. Therefore, comprehensive studies mapping the effects of Fingolimod on the brain are crucial to reveal its potential mechanism of action and suggest it as a potential therapeutic agent for other neurodegenerative diseases beyond MS. In this study, we have evaluated the effects of Fingolimod on the proteome of murine CB and FC. We have selected these brain regions as the CB is the main affected brain region in MS patients and the FC is also severely affected in MS patients and has been suggested to be associated with cognitive impairment in MS patients [16, 17, 18].

## 2 MATERIALS AND METHODS

### 2.1 Mice

Animal experiments were approved by the Macquarie University Animal Ethics Committee (AEC) in accordance with the Australian Code of Practice for the Care and Use of Animals for Scientific Purposes and the guidelines of the ARVO Statement for the use of animals in Ophthalmic and Vision Research. A total of 10 male 8‐week‐old, CBA/CaHArc mice were used in this study. Mice were randomly assigned into two groups comprising a Fingolimod treated group (*n* = 5) and an untreated control group (*n* = 5). All mice were housed under standard conditions at a 12‐h light/dark cycle with free access to food and water ad libitum for 2 weeks prior to the beginning of the study. The mice were treated for 2 months, all mice were anesthetized at the end of experiment using carbon dioxide and the brain regions including FC and CB were dissected and stored at −80°C after snap‐freezing in liquid nitrogen for further analysis.

### 2.2 Fingolimod treatment

Mice in treatment and control groups received either 5 mg/kg Fingolimod or vehicle control (saline) through weekly intraperitoneal (i.p.) injection. Mice were routinely monitored during the experiment for general well‐being [19].

### 2.3 Protein sample preparation

Brain tissue samples were lysed in lysis buffer containing 0.15 M NaCl, 1 mM EDTA, 1% Triton X‐100, and 1% Protease Inhibitor Cocktail followed by a sonication using probe sonicator (3 pulses/15 s/50 Hz with 20 s between each pulse) and centrifugation at 15,000 g for 10 min at 4°C. The supernatants were collected and transferred into a new tube and insoluble materials were removed. Concentration of the extracted proteins was measured using BCA assay kit (Pierce, Rockford, USA) with bovine serum albumin (BSA) as a standard, and 150 μg protein per sample was used for digestion. Protein reduction was performed by addition of 0.1 volume of 10 mM dithiothreitol (DTT) for 1 h at room temperature (RT), followed by alkylation with 0.05 volume of 50 mM iodoacetamide (IAA) for 1 h in the dark at RT. To quench the alkylation reaction, 5 mM DTT was added to the sample for 15 min in the dark.

Dual digestion was performed using Lys‐C (Wako, Japan) at a ratio of 1:100 enzyme/protein overnight at RT, followed by Trypsin (Promega, Madison, WI) digestion at a ratio of 1:100 enzyme/protein for 5 h at 37°C. Protein samples were acidified with TFA to final concentration of 1% (pH 2–3) and desalted by SDB‐RPS (3 M, Empore) Stage Tips.

**Significance Statement**

Fingolimod (FTY720) is a Food and Drug Administration (FDA) approved oral drug to manage multiple sclerosis (MS) symptoms. Fingolimod also showed beneficial effects against Alzheimer's (AD) and Parkinson's diseases (PD) pathologies but the mechanisms underlying its therapeutic effects are not well understood. Here, we used a tandem mass tag (TMT) isobaric labeling‐based quantitative mass‐spectrometric approach to investigate Fingolimod induced proteome changes in two brain regions that are severely affected in MS including the cerebellum (CB) and frontal cortex (FC). Our proteomic analysis yielded 2609 and 3086 differentially expressed proteins in CB and FC, respectively, between the Fingolimod treated and control groups. Further analysis using these DEPs identified metabolic reprogramming through upregulation of oxidative phosphorylation and downregulation of glycolysis and pentose phosphate pathway in both brain regions of Fingoimod treated group. In addition, an immunomodulation effect of Fingolimod was suggested through upregulation of retrograde endocannabinoid signaling and autophagy pathways and downregulation of neutrophil degranulation and IL‐12 mediated signaling pathway. These data supported that Fingolimod could be a potential and effective treatment against neurodegenerative diseases via modulating the metabolic reprogramming and neuroinflammation pathways.

### 2.4 TMT labeling of peptides, liquid chromatography tandem mass spectrometry analysis, and peptide to spectrum matching

**FIGURE 1:**
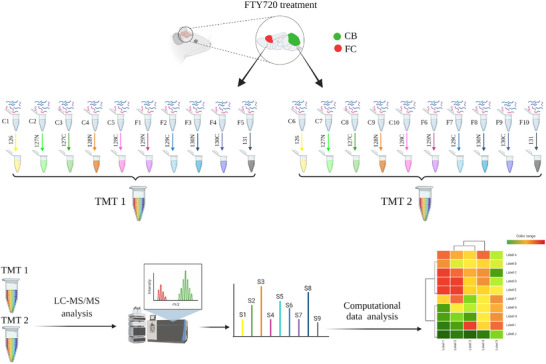
Experimental work flow of the study. CB and FC were dissected from FTY720 treated and control groups (*n* = 5 each). Proteins were extracted, reduced, alkylated, and digested with Lys‐C and trypsin. Labeling was performed using 10 plex TMT, then fractionated and analyzed by LC‐ESI‐MS/MS on a Thermo Scientific Orbitrap Fusion Mass Spectrometer (SPS‐MS3 method), and finally computational analyses were performed to identify the changes between FTY720 treated and control groups. CB, cerebellum; FC, frontal cortex; LC‐ESI‐MS/MS, liquid chromatography‐electrospray ionization tandem mass spectrometry; TMT, tandem mass tag

Two separate 10‐plex tandem mass tag (TMT) experiments were designed to accommodate 20 biological samples (5 FC and 5 CB each from both treated and control mice), as shown in Figure 1. The TMT labeling workflow was performed as previously reported [20]. Briefly, dried peptides were resuspended in 100 mM HEPES buffer (pH 8.2) and concentration was evaluated by MicroBCA protein assay kit (Thermo Scientific, Rockford, IL). A total of 150 μg peptides from each sample were labeled using 0.8 mg of TMT reagent (Thermofisher) for 1 h at RT with occasional vortexing. To quench the unbound TMT labels, 8 μl of 5% fresh hydroxylamine was added to each sample followed by additional vortexing and incubation for 15 min at RT. After the reaction, 10 respective labeled samples were pooled together and dried completely using a vacuum centrifugation. The labeled samples were reconstituted in 1% formic acid and desalted using Sep‐Pak C18 cartridges (Waters, Milford, MA, USA). Subsequent fractionation was performed using a high‐pH reversed phase fractionation (HpH), and then peptides were consolidated into 16 fractions. Fractionated peptide samples were reconstituted in 30 μl of 0.1% formic acid and 10 μl of samples were analyzed using an Orbitrap Fusion Tribrid‐MS (Thermo Scientific, USA) equipped with an ultra‐high pressure liquid chromatography system (Proxeon). Peptides were separated for 3‐h on a reverse phase column with a gradient of 6%–30% acetonitrile in 0.125% formic acid at a flow rate of ∼400 nl/min. In each data collection cycle, one full MS scan (400–1400 *m*/*z*) was acquired in the Orbitrap (120,000 resolutions at 400 *m*/*z* and an atomic gain control (AGC) of 2 × 10^5^). MS3 was performed using higher energy collisional dissociation (HCD) with 55% collision energy and reporter ion detection in the Orbitrap with an AGC of 150,000 ions, a resolution of 60,000 and a maximum ion accumulation time of 150 ms. Peptide fragmentation and collection of reporter ion spectra were performed using the synchronous precursor selection (SPS‐MS3 method) [21]. In this method, first MS2 analysis was conducted using CID fragmentation on the top 10 most intense ions with following settings: normalized collision energy (NCE) of 35%, AGC 4 × 10^3^, isolation window 0.5 Da, maximum ion accumulation time 150 ms with 40 s of dynamic exclusion. Following each MS2 scan, for the MS3 analyses, precursor isolation was performed using a 2.5 Da window and fragmented in the ion trap using CID as above, except with an AGC setting of 8000. Multiple fragment ions (SPS ions) were co‐isolated and further fragmented by HCD at NCE of 37.5%. Selection of fragment ions was based on the previous MS2 scan and the MS2‐MS3 was conducted using recently described sequential precursor selection (SPS) methodology [21]. In‐house software tools were used to convert RAW file to the mzxml format [22]. Correction of erroneous charge state and monoisotopic *m*/*z* values were performed using method detailed in Huttlin et al. [23]. Sequence assignment of MS/MS spectra were made with the Sequest algorithm using an indexed Mus Musculus Uniprot database prepared with forward and reversed sequences concatenated as per the target‐decoy strategy [22]. Data searches were conducted using cysteine carbamidomethylation and TMT on the peptide N‐termini and lysine residues as static modifications, oxidation of methionine as a dynamic modification, precursor ion tolerance of 20 ppm, and a fragment ion tolerance of 0.8 Da (for CID). Sequest matches were filtered using linear discriminant analysis to a false discovery rate (FDR) of 1% at the peptide level based on matches to reversed sequences, as previously reported [22]. The final peptide‐level FDR fell well below 1% (∼0.2% peptide level). A reductionist model was used for assignment of peptides to protein matches, where all peptides were explained using the least number of proteins. Protein rankings were generated by multiplying peptide probabilities and the dataset was finally filtered to 1% protein FDR.

Quantitation of peptides using TMT reporter ions was performed as previously published [22]. Briefly, a 0.003 Th window centered on the theoretical *m*/*z* value of each reporter ion was recorded for each of the 10 reporter ions, and the intensity of the signal closest to the theoretical *m*/*z* value was recorded. Peptides were only considered quantifiable if the total signal‐to‐noise (S/N) for all channels was >200 and a precursor isolation specificity of >0.75. Within each TMT experiment, reporter intensities values were normalized by summing the values across all peptides within each channel and then each channel was corrected so that each channel had the same summed value. Protein quantitation was performed by summing the normalized S/N values for all peptides assigned to a given protein.

### 2.5 Analysis of multiplexed quantitative proteomics data

The TMTPrepPro package [24, 25] was used to further analyze the identified proteins. The differentially expressed proteins (DEPs) were identified using Student's *t*‐test and fold change (FC) between treated and control groups in each brain region. The overall fold changes were calculated as means of the respective ratios. Two criteria were applied to determine significantly altered proteins: FC over 1.20 and *p*‐value less than 0.05 were considered for proteins increased in abundance and FC lower than 0.83 and *p*‐value less than 0.05 for proteins decreased in abundance [25, 26]. DEPs were subjected to pathway and biological process enrichment analyses using Enrichr and resulting pathways were also checked in DAVID, and in both databases KEGG pathways were selected [27, 28, 29]. Protein interaction networks were identified and visualized using the STRING plugin in Cytoscape (https://cytoscape.org) based on pathways and biological processes with confidence cutoff of 0.5 [30]. Subsequent analysis of the protein–protein interaction (PPI) network was performed using the Maximal Clique Centrality (MCC) algorithm in the CytoHubba plugin to identify hub genes within the network [31].

## 3 RESULTS

### 3.1 Fingolimod induced significant proteome changes in both cerebellum and frontal cortex regions

**FIGURE 2:**
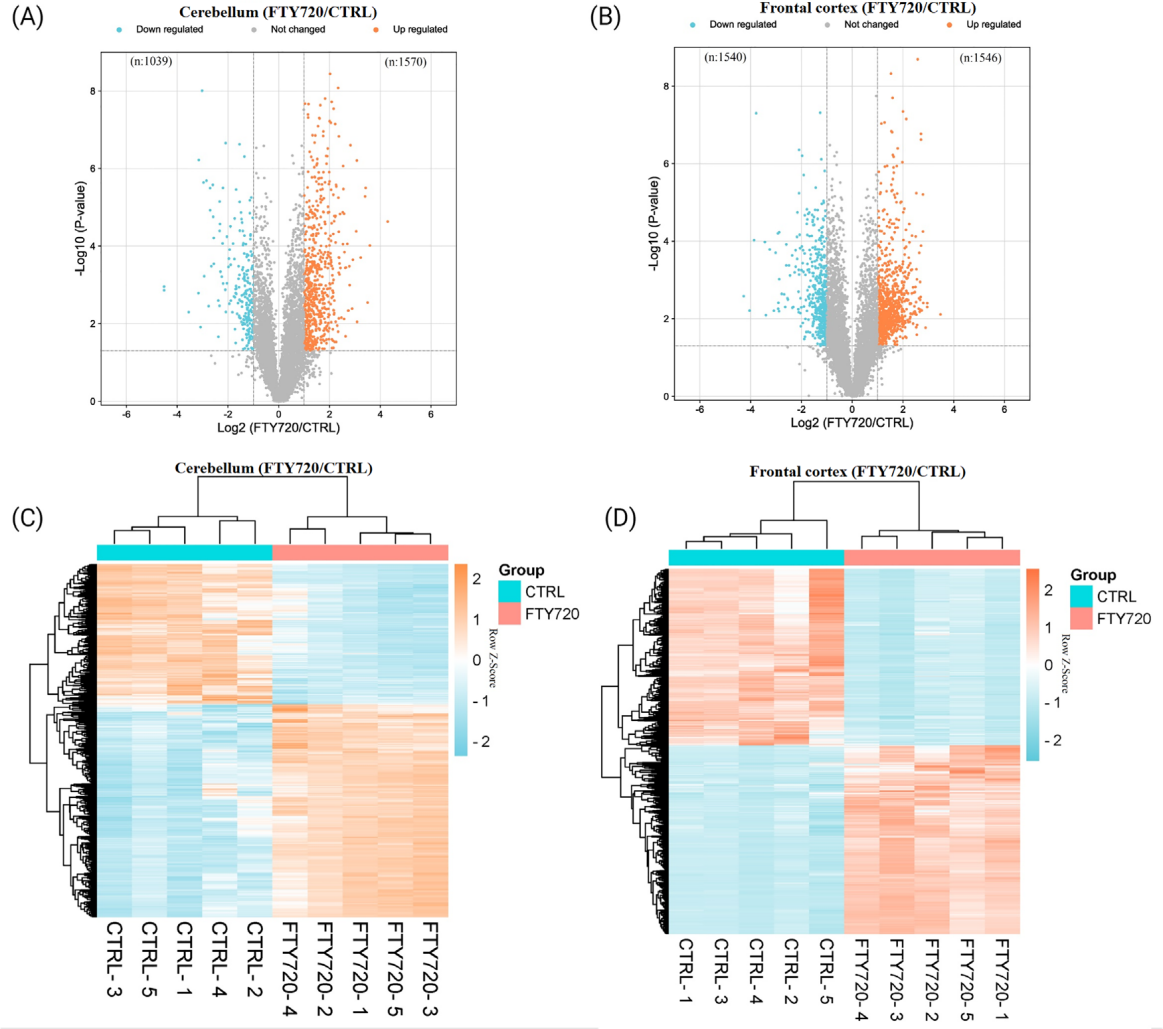
Volcano plots (A) and (B) indicating differentially expressed proteins in cerebellum and frontal cortex of FTY720 treated group compared to control group; the x‐axis represents log2 fold change in abundance in FTY720 treated group versus control group, while y‐axis represents −Log10 *p*‐value. Heatmaps (with hierarchical clustering) (C) and (D) showing the log‐transformed ratios of differentially expressed proteins from CB and FC samples respectively (FTY720 treated vs. control). CB, cerebellum; FC, frontal cortex

**FIGURE 3:**
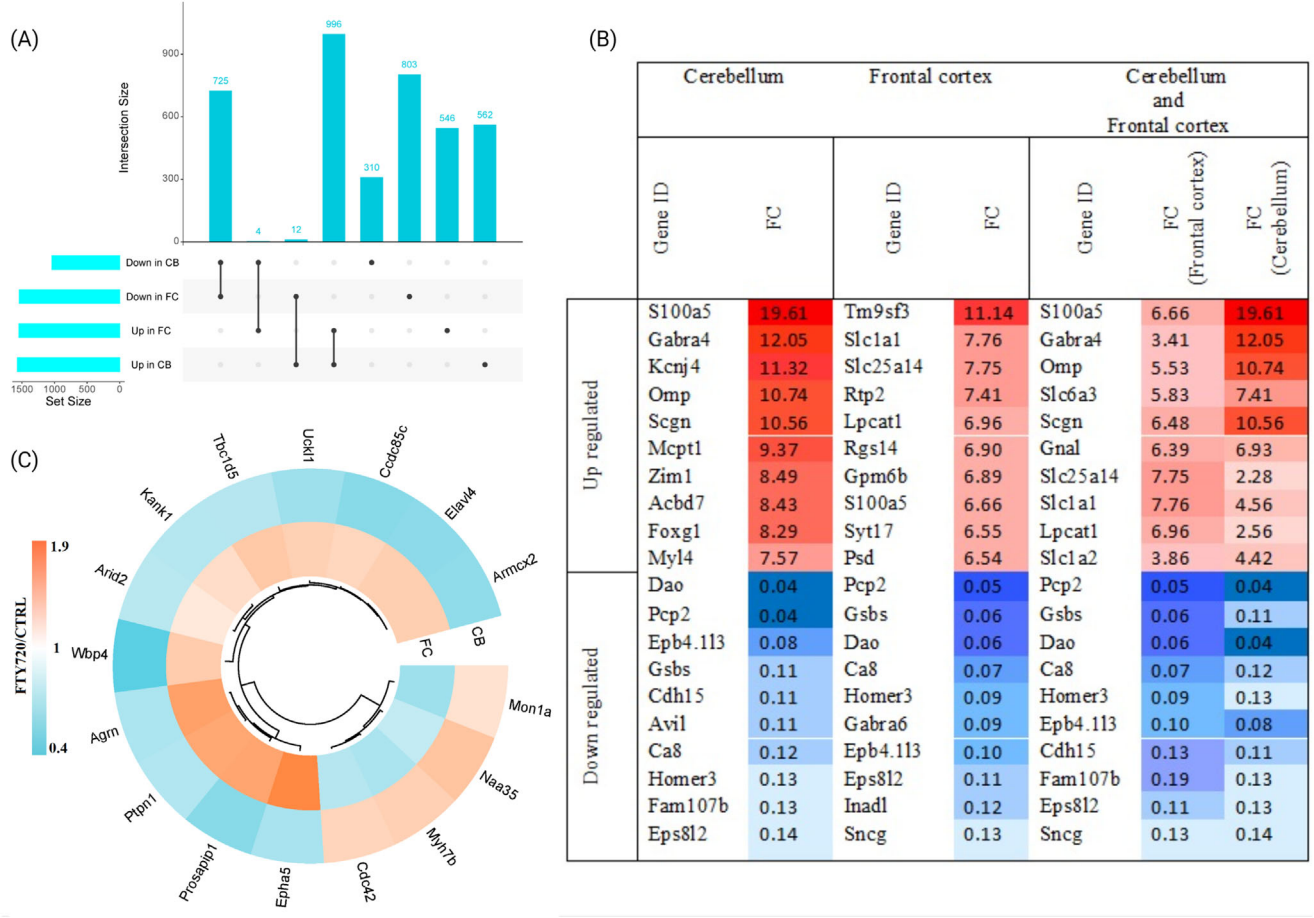
(A) Upset plot indicating the overlap of proteins with either increased or decreased abundance in CB and FC. (B) Top 10 most significantly altered proteins increased and decreased in abundance in each brain regions and also top 10 proteins with increased and decreased abundance shared between CB and FC. (C) Proteins with different alteration pattern between FC and CB. FC, frontal cortex; CB, cerebellum

A total of 6749 proteins were identified in CB and 6319 proteins were identified in FC at a protein FDR ˂1% (Supplementary File 1). Comparing the proteome between Fingolimod treated and control groups based on the defined fold changes (log2 FC (±0.26)) and statistical significance (*p* value less than 0.05) returned 1039 proteins decreased in abundance and 1570 proteins increased in abundance in CB, and 1540 proteins decreased in abundance and 1546 proteins increased in abundance in FC (Figure 2A, B). Hierarchical clustering analysis of DEPs of each brain region depicted the overall consistency of proteome changes across biological replicates in both Fingolimod and control groups, which confirmed the reproducibility of the data (Figure 2C, D). The presence of DEPs in each brain region were investigated and results showed that 725 proteins decreased in abundance and 996 proteins increased in abundance were common between two brain regions (Figure 3A). Top 10 significantly altered proteins in each brain region and shared between CB and FC are shown in Figure 3B. Furthermore, four proteins were decreased in abundance in CB and increased in abundance in FC, while 12 proteins were increased in abundance in CB and decreased abundance in FC (Figure 3C).

### 3.2 Pathway classification of the differentially expressed proteins

**FIGURE 4:**
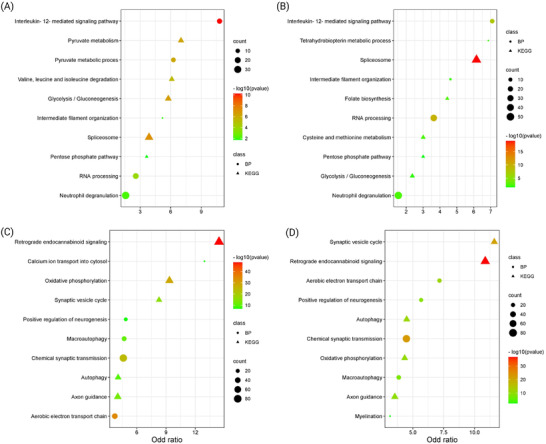
Top 5 most significantly altered downregulated pathways and biological processes in CB (A) and FC (B), and top 5 most significantly altered upregulated pathways and biological processes in CB (C) and FC (D). CB, cerebellum; FC, frontal cortex

The DEPs from CB and FC were subjected to biochemical pathway enrichment and functional protein network analyses to reveal potential molecular mechanisms underpinning beneficial effects of Fingolimod for neurodegenerative diseases particularly MS. A literature‐based investigation was also performed to select relevant pathways related to neurodegenerative diseases with a special focus on MS, AD, and PD. Taken together, our results revealed several pathways and biological processes impacted by Fingolimod; we subsequently used a hierarchical clustering tree to cluster pathways/biological processes with many shared genes together to identify pathways/biological processes from each group that were relevant to neurodegenerative diseases. The top 5 most significant pathways and the top 5 significant biological processes are shown in Figure 4 (Supplementary File 2). Interestingly, Fingolimod affected both brain regions in a similar fashion and most of the pathways were commonly affected within both brain regions. However, there were some minor differences such as upregulation of Myelination and downregulation of spliceosome related proteins in FC but not CB as well as downregulation of metabolic pathways in CB compared to FC, such as aspartate and glutamate metabolism and valine, leucine, and isoleucine amino acid degradation networks. Retrograde endocannabinoid (eCB) signaling, oxidative phosphorylation, synaptic vesicle formation, axonal guidance, and autophagy pathway downregulations have been repeatedly reported in neurodegenerative diseases. The top 5 most significantly altered upregulated pathways and biological processes between both the brain regions are shown in Figure 4C, D. On the other hand, downregulation of glycolysis/gluconeogenesis, pentose phosphate pathway, neutrophil degranulation, and interleukin 12 (IL‐12) mediated signaling were among the top most significantly altered downregulated pathways and biological process between both brain regions (Figure 4A, B).

### 3.3 Protein–protein interaction analysis

**FIGURE 5:**
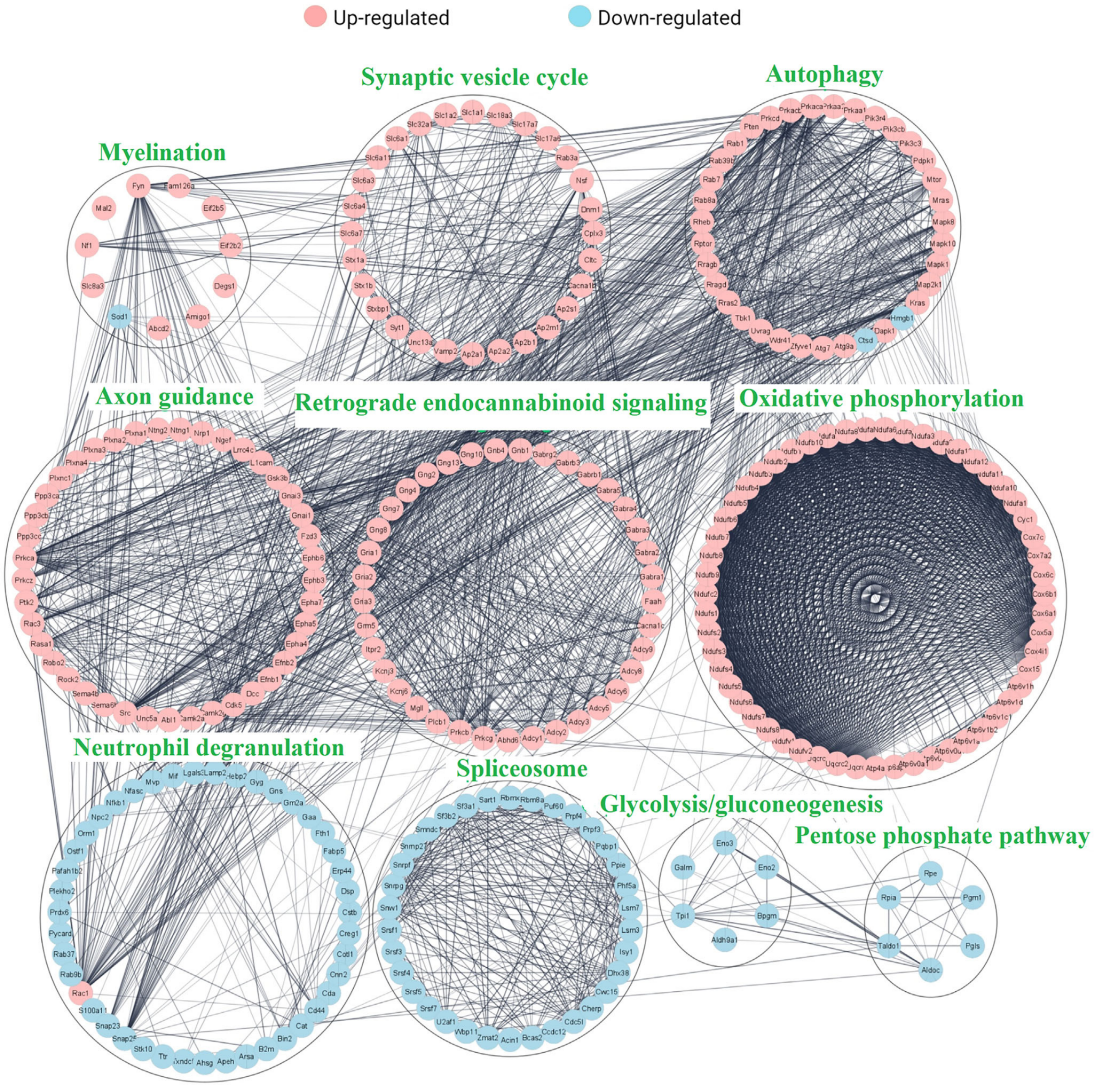
Protein–protein interaction between top enriched up (red) and down (blue) regulated pathways

**FIGURE 6:**
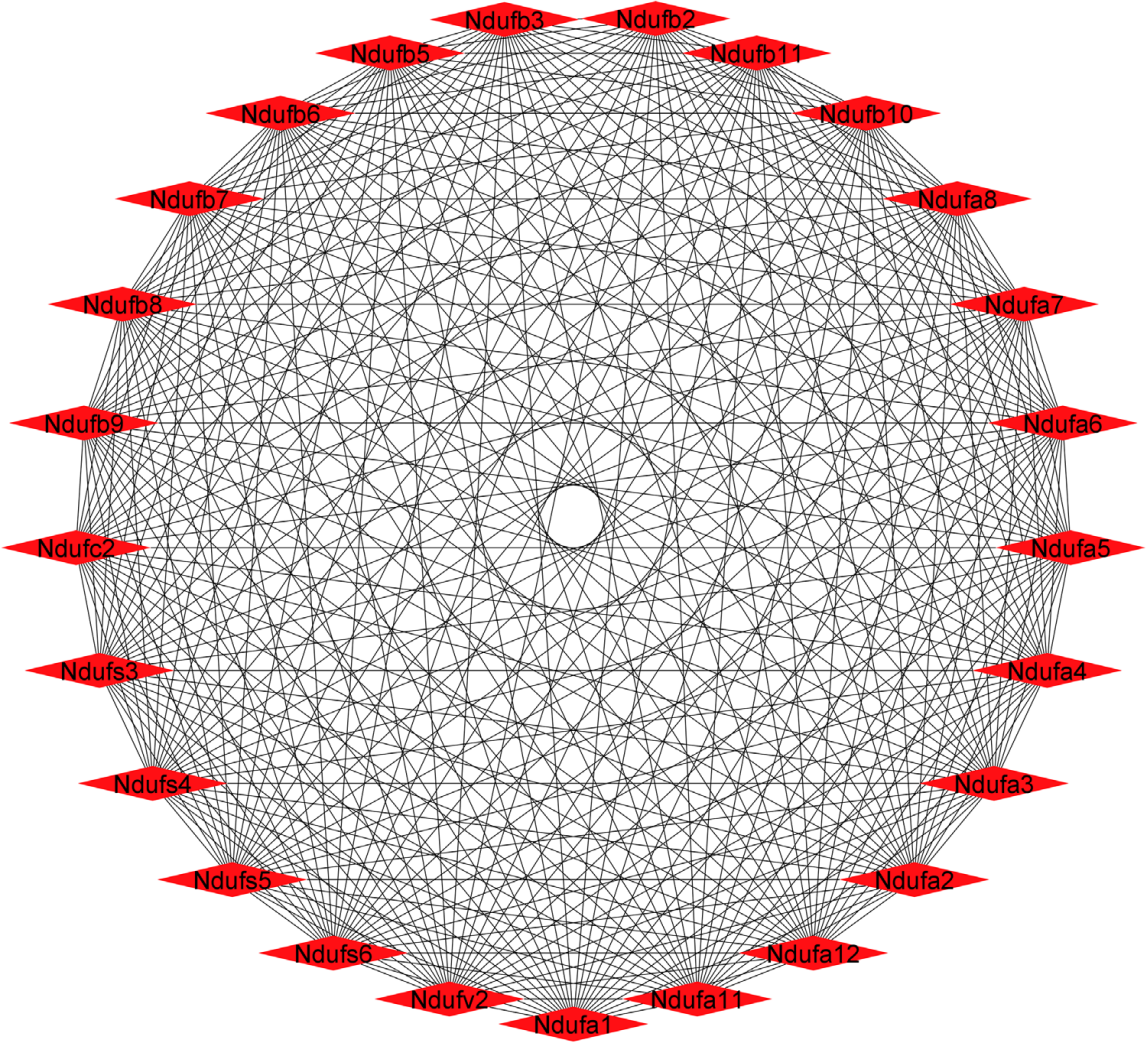
Top ranked hub genes based on the Maximal Clique Centrality (MCC) algorithm in cytoHubba plugin of Cytoscape

For PPI analysis, we analyzed all genes of DEPs within the top five most significantly altered enriched KEGG pathways and biological processes in each brain region to avoid missing potential interactions. Retrograde eCB signaling and oxidative phosphorylation shared 33 DEPs that are shown within the retrograde eCB signaling in the PPI network in Figure 5. In addition, when two pathways shared multiple genes, the pathway with higher number of genes appears in the network. PPI of the DEPs within the network are shown through circular layouts in Figure 5. Subsequent analysis of the PPI network using the MCC algorithm in the CytoHubba plugin returned 25 genes as the first ranked hub genes. Interestingly all of these genes were related to complex I of the oxidative phosphorylation chain (Figure 6).

### 3.4 Upregulation of retrograde endocannabinoid signaling and oxidative phosphorylation pathways

**FIGURE 7:**
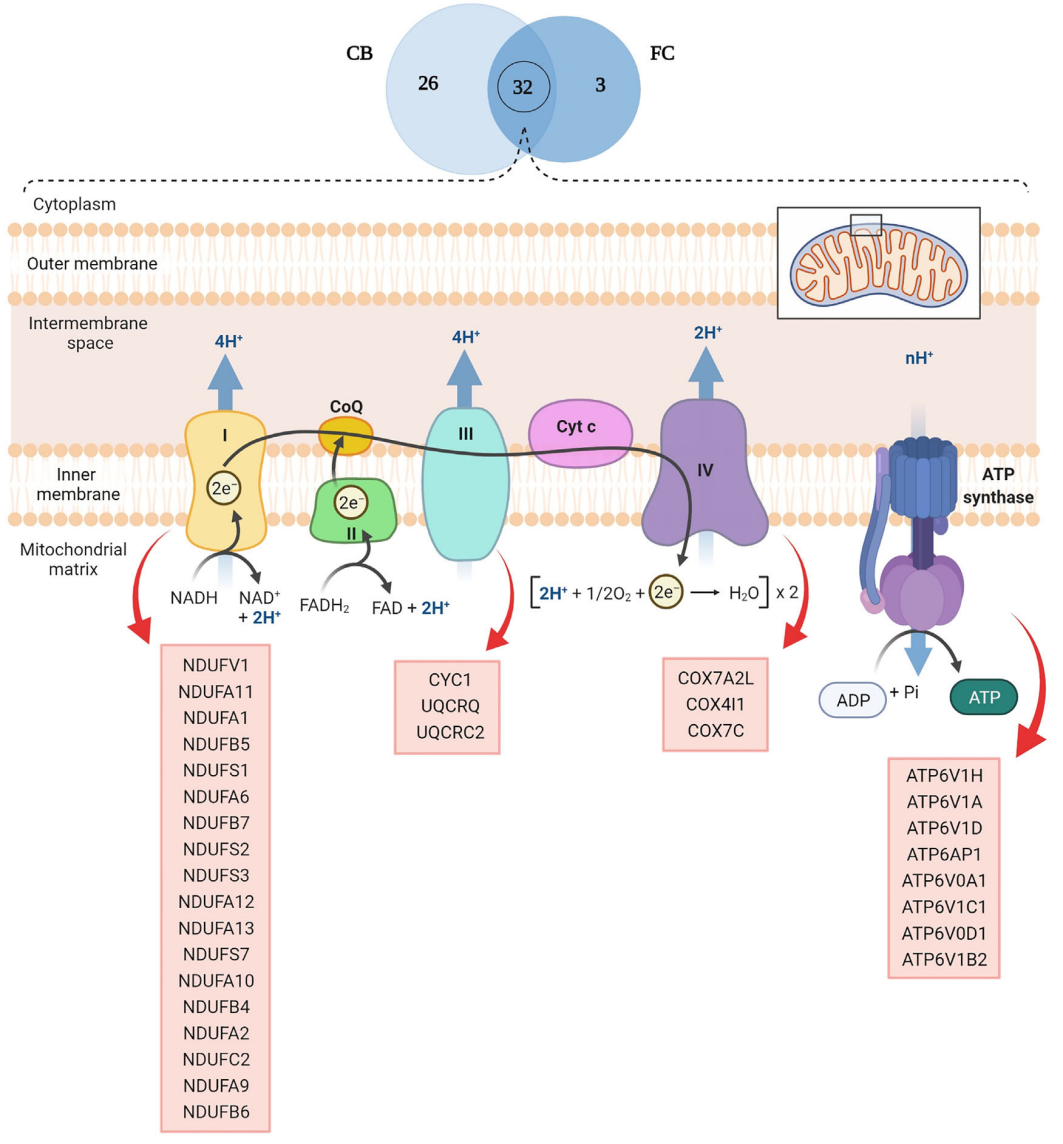
Thirty‐two proteins decrease in abundance involved in oxidative phosphorylation that were common between CB and FC, and their localization in each complex of the mitochondrial oxidative phosphorylation chain. CB, cerebellum; FC, frontal cortex

Retrograde eCB signaling, one of the key pathways affected in various neurodegenerative diseases, was enriched and showed 69 proteins increased in abundance in FC and 80 proteins increased in abundance in CB. It was the most significantly enriched pathway in both brain regions based on resultant *p*‐value. Of these, 58 proteins were shared between the two brain regions. In addition to oxidative phosphorylation, another commonly impaired pathway in neurodegenerative disease was enriched by 35 and 58 upregulated proteins in FC and CB, respectively. Thirty‐two of these proteins were shared between two brain regions, and complex I oxidative phosphorylation chain was the most affected complex with 18 proteins increasing in abundance, followed by complex V with 8 and IV and III with 3 altered proteins each (Figure 7).

### 3.5 Upregulation of autophagy and axonal guidance pathways

Autophagy was also a significantly upregulated common pathway between the two brain regions. FC showed 37 DEPs related to autophagy pathway, while CB showed 35 such DEPs. PPI analysis revealed a strong interaction between autophagy and axon guidance associated network, another upregulated pathway in both brain regions. Axon guidance was enriched by 36 DEPs in CB and 41 DEPs in FC. PPI analysis indicated a close relationship between these two pathways (Figure 5).

### 3.6 Downregulation of biological process related to neuroinflammation

**FIGURE 8:**
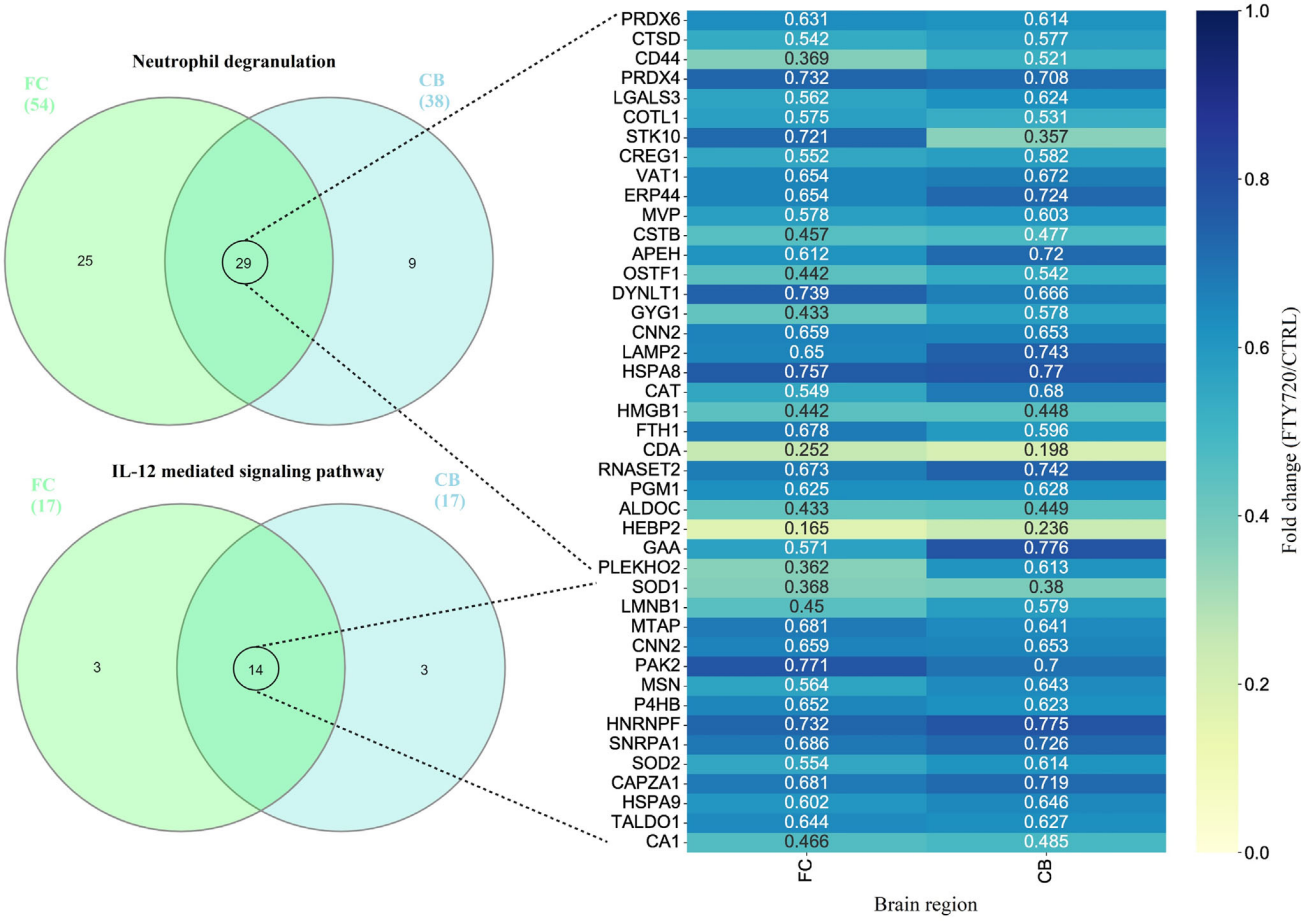
Downregulated proteins involved in neutrophil degranulation and IL‐12 mediated signaling pathways that were common between FC and CB. CB, cerebellum; FC, frontal cortex; IL‐12, interleukin 12

Amongst the top 5 downregulated biological processes, neutrophil degranulation was enriched by 54 and 38 DEPs in FC and CB, respectively, and the IL‐12 mediated signaling pathway was enriched by 17 DEPs in both brain regions. This indicated the potential anti‐inflammatory effects of Fingolimod. Of these proteins, 29 DEPs from neutrophil degranulation and 14 proteins from IL‐12 mediated signaling pathway were common between both brain regions (Figure 8)

### 3.7 Alteration of known biomarkers and biological process related to MS

Results of our study showed a decrease in abundance of neurofilament light chain (NfL) in both brain regions. Increased serum level of NfL is known as an important biomarker of axonal degeneration in MS patients. In addition, upregulation of myelination was also evident in FC with 10 DEPs but not in CB. Furthermore, our results did not show any significant changes in level of S1PR1 in CB, while show an increase in FC, S1PR1 is currently known as a main target of Fingolimod (Supplementary File 1).

## 4 DISCUSSION

Fingolimod is an FDA approved drug for MS; however, its possible beneficial effects against other neurodegenerative disease conditions have also been reported [13]. Although, a growing number of studies have been performed to investigate the mechanisms underlying therapeutic effects of Fingolimod on MS and other neurodegenerative diseases, much remains unknown in terms of downstream molecular effects of the drug in the brain [13, 32]. This study aimed to investigate the proteome changes in two mouse brain regions, CB and FC following treatment with Fingolimod to reveal affected biological pathways that may drive its therapeutic effects. These two brain regions were selected since they are severely affected in MS. Our proteomics approach identified a greater number of proteins in CB with 6749 proteins compared to FC with 6319 proteins. The comparison between Fingolimod treated and control groups showed a larger proportion of DEPs in FC with 3086 DEPs compared to CB with 2609 DEPs. Further analyses revealed common and distinct altered biological pathways in these brain regions, such as upregulation of retrograde eCB signaling, oxidative phosphorylation, autophagy and synaptic vesicle cycle, and downregulation of neutrophil degranulation, Il‐12 mediated signaling pathway, and glycolysis/gluconeogenesis. In addition, upregulation of the myelination pathway was observed in FC, but not in CB. Furthermore, our results also provided validation of several protein markers such as NfL, that are used for diagnosis of MS and other neurological disorders [33].

Retrograde eCB signaling is one of the major altered pathways in several neurodegenerative diseases such as AD, PD, and MS [34, 35, 36]. Recently a meta‐analysis on transcriptomics datasets from five brain regions of AD patients including hippocampus, CB, temporal cortex, entorhinal, and FC highlighted retrograde eCB signaling as a key altered pathway in AD patients [37]. Retrograde signaling is the main mechanism by which eCBs regulate short‐ and long‐term forms of plasticity at both excitatory and inhibitory synapses [35]. Retrograde eCB signaling has also been implicated in modulation of neuroinflammation [38]. Neuroinflammation is an important contributor to pathogenesis of AD, PD, and MS; therefore, immunomodulation has emerged as a potential strategy to combat these disorders [35, 39, 40]. Anti‐inflammatory potential of Fingolimod has been previously reported in different animal models; however, the downstream biochemical networks have remained largely poorly understood [41, 42]. The eCB system has been shown to activate anti‐inflammatory signaling pathways that modulate immune cell functions; such as modulation of microglial activation in different neurodegenerative diseases [43]. Currently cannabinoid‐based drugs are being used to treat symptoms of several neurodegenerative diseases particularly MS [35, 44]. Interestingly, cannabinoids also induce autophagy in an AMPK‐dependent manner, via their anti‐inflammatory actions [45]. Autophagy is essential for healthy aging of neurons and impaired autophagy has been widely reported in neurodegenerative diseases leading to accumulation of abnormal proteins in these diseases [46]. A possible mechanism by which autophagy reduces inflammation is through degradation of the inflammasome, resulting in decreased levels of IL‐1β secretion [47]. Moreover, clearance of damaged organelles, protein aggregates, and apoptotic bodies through autophagy may ameliorate cell stress and prevent secondary necrosis and amplification of the inflammatory response [48, 49]. Mounting evidence has also indicated that autophagy plays an essential role in axonal growth and guidance [50, 51]. Axonal guidance proteins guide axonal growth during development and control structural plasticity of synaptic connections in adults. Any alteration in their expression or function may induce pathological changes in neural circuits that may predispose the cells to neurodegenerative changes [52]. Axonal degeneration has been reported to precede neuron death and clinical symptoms of neurodegenerative diseases, therefore inhibiting axonal loss or promoting its regeneration has become a promising approach against these devastating diseases prior to the onset of clinical symptoms [53]. In this regard, Fingolimod has been previously reported to increase neurite growth and axonal regeneration of both peripheral and CNS neurons [54].

Another upregulated pathway in both the brain regions is oxidative phosphorylation particularly complex I of the mitochondrial electron transport chain. Protective effects of Fingolimod on mitochondria have been reported previously [41]. Recent studies have indicated that restoration of mitochondrial function through therapeutic agents, antioxidants or physical exercise can delay the development of neurodegenerative diseases [55, 56, 57]. It should be noted that the retrograde eCB signaling and oxidative phosphorylation pathways share several proteins which may suggest their close interaction.

Interestingly, our results showed a metabolic reprograming upon treatment with Fingolimod as increased oxidative phosphorylation was associated with decreased glycolysis. Energy reprogramming reported to be involved during inflammation and exposure to pro‐inflammatory stimuli may induce a metabolic switch from oxidative phosphorylation to glycolysis in macrophages [58, 59]. Such a phenomena has also been reported in microglia in neuroinflammatory conditions, which is beneficial for generation of intermediates for cell growth and cytokine production [58]. Interestingly, blocking glycolysis also has been shown to reprogram microglia back to mitochondrial oxidative phosphorylation from glycolysis, which has been shown to be associated with reduced light‐induced retinal neurodegeneration in a mouse model [60]. Intriguingly, increased glycolysis has also been suggested as a compensatory response to decreased glucose levels and indicated a possible protective mechanism against neurodegeneration [61].

Our results also showed downregulation of pathways that are involved in neuroinflammation such as neutrophil degranulation and IL‐12 mediated signaling pathway. Increased neutrophil degranulation is a common feature of many inflammatory diseases, and has also been reported in patients with neurodegenerative diseases [62, 63, 64]. The neuroinflammatory IL‐12 signaling pathway was also shown to be a key pathway in neurodegenerative diseases, particularly in AD, as it impairs oligodendrocyte survival and neuronal homeostasis [65]. Interestingly, lack of IL‐12 signaling pathway did not change inflammatory gene expression in a mouse model of AD but did reverse the loss of mature myelin‐producing oligodendrocytes, as well alterations in neuronal homeostasis [65].

Our study also highlighted upregulation of myelination related proteins in FC, but not CB, of Fingolimod treated group compared to control group. While some studies have indicated the cerebellar remyelination capability of Fingolimod [66, 67, 68, 69], others observed no significant remyelination in mice treated with Fingolimod after cuprizone‐induced demyelination [70, 71]. Our results did not show an upregulation of the remyelination pathway in CB of mice, although it should be noted that we have investigated the effect of fingolimod treatment on healthy mice only. In this regard, upregulation of PPAR signaling pathway has also been observed only in FC but not CB, this pathway has been shown to promote myelinogenesis, antioxidant defenses, and mitochondrial respiratory activity [72]. The mechanisms by which PPAR signaling promotes remyelination in MS may be through downregulation of NF‐κB/β‐catenin and upregulation of PI3K/Akt pathways [72, 73]. Neuroprotective effects of fingolimod following traumatic brain injury (TBI) and other neurodegenerative disease has also been shown to be correlated with activation of the PI3K/Akt pathway [74, 75]. Lysosome associated protein networks were also differentially affected in each brain region, while it was upregulated in CB, its downregulation was evident in FC. Previous studies indicated that lysosomes contribute to myelin sheath degeneration and lysosomal swelling has been reported in proximity to degenerated astrocytes [76]. In addition, our results showed upregulation of proteins involved in sphingolipid metabolism including Degs1, Smpd3, and Sphk1 in both brain regions and Smpd1 only in FC. These results are in contrast to previous reports indicating that mice lacking Smpd1 had increased oligodendrocyte cell count and faster remyelination than wild type mice after demyelination by cuprizone [77].

Another interesting result of our study was the downregulation of NfL. Increased level of NfL in cerebrospinal fluid and serum of amyotrophic lateral sclerosis, AD, and MS patients is considered as a promising biomarker of axonal injury [78, 79]. In conclusion, this study revealed diverse molecular pathways and proteins that are affected by Fingolimod in CB and FC, with most of these changes occurring in both of these two brain regions. Energy reprogramming and neuroinflammation modulation were evident as two major mechanisms underpinning therapeutic effects of Fingolimod on neurological disorders. This information will provide a foundation for future clinical and animal studies and lead to a better understanding of this therapeutic agent and its potential application for other neurological disorders.

## CONFLICT OF INTEREST

The authors declare no conflict of interest.

## Supporting information

Supplementary information

Supplementary information

## Data Availability

The mass spectrometry proteomics data have been deposited to the ProteomeXchange Consortium via the PRIDE partner repository with the dataset identifier PXD034427.
